# Clinical Analysis of Algerian Patients with Pompe Disease

**DOI:** 10.1155/2017/9427269

**Published:** 2017-02-06

**Authors:** Y. Sifi, M. Medjroubi, R. Froissart, N. Taghane, K. Sifi, A. Benhabiles, S. Lemai, S. Semra, H. Benmekhebi, Z. Bouderda, N. Abadi, A. Hamri

**Affiliations:** ^1^Service de Neurologie, Faculté de Médecine, Université Constantine 3, Constantine, Algeria; ^2^Laboratoire de Biologie et de Génétique Moléculaire, Faculté de Médecine, Université Constantine 3, Constantine, Algeria; ^3^Service de Pédiatrie, Faculté de Médecine, Université Constantine 3, Constantine, Algeria; ^4^Laboratoire des Maladies Héréditaires du Métabolisme, Biochimie et Biologie Moléculaire, Centre de Biologie et Pathologie Est, Hospices Civils de Lyon, Bron, France; ^5^Laboratoire de Biochimie, Faculté de Médecine, Université Constantine 3, Constantine, Algeria; ^6^Service de Chirurgie Orthopédique, Faculté de Médecine, Université Constantine 3, Constantine, Algeria; ^7^Service Médecine Physique et Réadaptation Fonctionnelle, Faculté de Médecine, Université Constantine 3, Constantine, Algeria; ^8^Service de Réanimation Médicale, Faculté de Médecine, Université Constantine 3, Constantine, Algeria

## Abstract

Pompe's disease is a metabolic myopathy caused by a deficiency of acid alpha-glucosidase (GAA), also called acid maltase, an enzyme that degrades lysosomal glycogen. The clinical presentation of Pompe's disease is variable with respect to the age of onset and rate of disease progression. Patients with onset of symptoms in early infancy (infantile-onset Pompe disease (IOPD)) typically exhibit rapidly progressive hypertrophic cardiomyopathy and marked muscle weakness. Most of them die within the first year of life from cardiac and/or respiratory failure. In the majority of cases of Pompe's disease, onset of symptoms occurs after infancy, ranging widely from the first to sixth decade of life (late-onset Pompe's disease or LOPD). Progression of the disease is relentless and patients eventually progress to loss of ambulation and death due to respiratory failure. The objective of this study was to characterize the clinical presentation of 6 patients (3 with EOPD and the other 3 with LOPD) of 5 families from the East of Algeria. All our patients were diagnosed as having Pompe's disease based on biochemical confirmations of GAA deficiency by dried blood spots (DBS) and GAA gene mutations were analyzed in all patients who consented (*n* = 4). Our results are similar to other ethnic groups.

## 1. Introduction 

Pompe's disease (PD), or glycogen storage disease type II (OMIM 232300) is an autosomal recessive disorder caused by a deficiency in the activity of the lysosomal enzyme acid alpha-glucosidase (GAA; EC.3.2.1.20), an enzyme that degrades lysosomal glycogen. As a result, glycogen accumulates in lysosomes of many types of cells but accumulates predominantly in skeletal muscle fibers. The process is progressive and finally destroys the muscle architecture and function [1–4]. The incidence of PD varies in different ethnic groups and for the different clinical forms. The rapidly progressive infantile-onset form has estimated frequencies of 1 : 138,000 in the Caucasian population [5], 1 : 50,000 in Taiwanese [6], and 1 : 31,000 in those of African ancestry [7]. The clinical presentation of PD variates according to the age of onset and rate of disease progression. The classic infantile form or infantile onset Pompe disease (IOPD) is characterized by progressive cardiac hypertrophy and rapid loss of muscle function. Symptoms are manifested shortly after birth and patients usually die within the first year of life [1, 7, 8]. In the majority of cases, onset of symptoms occurs after infancy, ranging widely from the first to sixth decade of life; this form is called late-onset Pompe's disease (LOPD). The progression of the disease in this form is relentless and patients eventually lose ambulation and then die due to respiratory failure [9, 10]. This phenotypic variation is associated with different levels of residual GAA activity; indeed GAA activity in infantile onset Pompe's disease (IOPD) is less than 1% of controls and is generally higher in (LOPD) [11–13]. The GAA gene (OMIM number 606800) has been localized to chromosome 17q25, 3 and a large number of mutations have been described to date, the majority being private. c-32-13 T>G mutation affects about 40% of alleles in late-onset phenotypes [14, 15]. GAA mutation is not required for the diagnosis of (PD), but it is important for prenatal diagnosis (especially in IOPD families). Until recently there was no therapy for patients with PD other than supportive care. This has changed and the prognosis of PD has been modified considerably by enzyme replacement therapy (ERT) [16–18], but at least one-third of children do not respond to ERT and ultimately die or do not reach a normal motor milestone [19]. The diagnosis of PD is based on a low level of GAA confirmed through a quantitative enzyme (GAA) activity assay in tissue samples, including blood, skin fibroblasts, and muscle. Historically, skin fibroblasts and muscle biopsy have been the samples of choice for measuring GAA activity; both require invasive procedures and take several weeks to obtain results. New GAA enzyme activity assays using blood samples, including dried blood spot (DBS), are rapidly being adopted because of their accuracy, speed, cost, and convenience [8, 20, 21]. The aim of our study was to report the phenotype of six patients from 5 families with PD, all from the East of Algeria.

## 2. Patients and Methods Study Design

The names of patients diagnosed with PD during the period from 2001 to 2013 were recruited from our centre of neurology and from the pediatric centre in Constantine. Informed consent has been signed by all patients or by their parents. Detailed pedigree charting was done and first degree relatives were examined. Patients were diagnosed as having PD based on biochemical confirmations of GAA deficiency by dried blood spots (DBS) (Centre de Référence des Maladies Héréditaires du Métabolisme, Hôpital Debrousse, Hospices Civils, Lyon, France). GAA gene mutations were analyzed in all patients who consented (*n* = 4) (Centre de Référence des Maladies Héréditaires du Métabolisme, Hôpital Debrousse, Hospices Civils, Lyon, France). Cross-reactive immunologic material or CRIM status was not determined. All patients provided a detailed medical history and underwent a clinical neurological examination. Motor performance was assessed using manual muscle testing (MMT). Serum creatine kinase (CK), serum glutamic oxaloacetic transaminase/aspartate transaminase (SGOT/AST), and serum glutamic pyruvic transaminase/alanine transaminase (SGPT/ALT) levels were determined during the initial presentation. One muscle biopsy was performed. Electrocardiography (ECG) and echocardiography as lung function tests were done on all patients. Our patients were treated with 20 mg/kg IV alglucosidase alfa (Myozyme®, provided by Genzyme Corporation) biweekly according to previously published reports [22–25]. Of these patients who were diagnosed to have PD, three were of the IOPD variety and the three other of the LOPD category. The clinical profile of these cases is as shown in Table 1.

## 3. Results

### 3.1. Late-Onset Pompe's Disease Cases

#### 3.1.1. Patient Number 1

**Figure 1:**
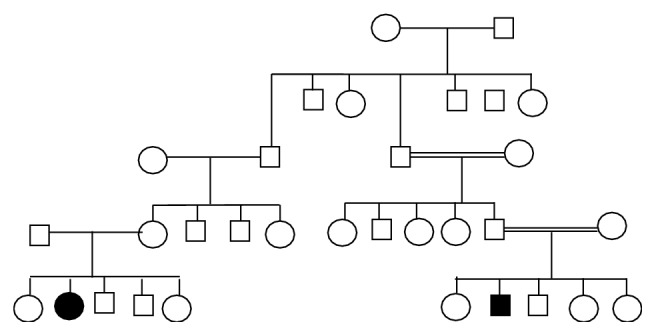
Family tree of patient number 1.

Patient number 1 is a 31-year-old man who developed since the age of 12 years a progressive pelvic muscle weakness responsible for difficulty in walking and climbing stairs; this was associated with a proximally predominant severe weakness of upper limb muscles at the age of 20 years. At that time a diagnosis of limb-girdle muscular dystrophy was then made. He was 23-year-old when he was seen again for exertional dyspnea and headaches when waking. Neurological examination revealed lumbar hyperlordosis with a waddling gait, a severe proximal weakness of lower limbs, upper limbs, and paraspinal muscles and amyotrophy in four limbs with moderate calves' hypertrophy. Only the knee reflexes were absent. He was at grade 3 of Vignos and Walton scales. ECG and echocardiography were normal; however lung function tests showed restrictive changes. Serum CK activity was 4 times the normal value and AST and ALT were 3 times the normal value. EMG showed myopathic changes, and a muscle biopsy revealed a dystrophic pattern. The activity of lysosomal acid maltase (acid alpha-glucosidase GAA) measured in leukocytes was reduced to about 6% of control values. Genetic studies revealed the presence of the mutation P. Tyr543Asp or p. y543D (c. 1627T>G) in the exon 11, that has never been described. Our patient was treated since 2007 with IV alglucosidase alfa (Myozyme), biweekly. But he had become wheelchair-dependent at the age of 27 years. The family history of the patient number 1 revealed one affected parental cousin who died from respiratory problems at 2008. The family pedigree is shown in Figure 1.

#### 3.1.2. Patient Number 2

Patient number 2 is the paternal cousin of patient number 1; she was 27-year-old when she was first examined in our centre. The age of onset has been estimated at 21 years with progressive difficulty in walking and climbing stairs. Two years later respiratory symptoms were reported and dyspnea worsened when she was supine. She became wheelchair bound at 25 years. The neurologic examination indicated severe proximal predominant muscles weakness in four limbs causing the patient's dependence on a wheelchair, a severe kyphoscoliosis with truncal muscle weakness, contracture of knee and ankles articulations, and amyotrophy in four limbs. Deep tendon reflexes were absent. She was at grade 10 of Vignos scale and 7 of Walton scale. EMG was compatible with a myopathic disorder. Serum CK activity was 6 times the normal value and AST and ALT were 4 times the normal value. ECG and echocardiography showed no cardiac abnormalities but lung function's tests showed restrictive changes. The activity of lysosomal acid maltase (acid alpha-glucosidase GAA) measured in leukocytes was very reduced. She did not benefit of genetic study. She was treated since 2007 with IV alglucosidase alfa (Myozyme), biweekly. She died 8 months later due to respiratory failure following respiratory tract infections.

#### 3.1.3. Patient Number 3

Patient number 3 is a 35-years-old man; he was 23-year-old when he was first examined in our centre. The age of onset has been estimated at 5 years with a progressive pelvic muscle weakness responsible for difficulty in walking. Neurological examination found a severe weakness and proximal amyotrophy of lower limbs with a discreet weakness of upper limb muscles. He was at grade 3 of Vignos and Walton scales. CK rate was 5 times the normal value and AST and ALT were normal. EMG was compatible with a myopathic disorder. ECG, echocardiography, and lung function tests were normal. The activity of lysosomal acid maltase measured in leukocytes was significantly decreased and the genetic studies revealed c.-32-13T>G homozygous mutation in the intron 1. He was treated since 2013 with IV alglucosidase alfa (Myozyme), biweekly. At the moment he is still ambulatory and able to climb stairs.

### 3.2. Infantile Pompe's Disease Cases

#### 3.2.1. Patient Number 4

A 5-month-old boy was born at full term. His family history revealed that an older sister died of hypertrophic cardiomyopathy with congestive heart failure at the age of four months. The child was sent to the pediatric centre because of a cardiac insuffisance; on examination, he had respiratory distress and tachycardia. There was marked hepatomegaly but no splenomegaly. A generalized hypotonia, decreased deep tendon reflexes, macroglossia, and swallowing disorders were found on neurological examination. Chest films showed cardiomegaly with a cardiothoracic ratio of about 0, 68%. The echocardiography demonstrated biventricular and interventricular hypertrophy but no left ventricular outflow tract obstruction. CK rate was 7 times the normal value and AST and ALT were 2 times the normal value. Quantitative blood alpha-glucosidase's level was null. He did not benefit of genetic study. The child was treated since 2012 with IV alglucosidase alfa (Myozyme), biweekly with medical treatment of the cardiomyopathy. After 06 months of treatment we noted stabilization of cardiomyopathy and swallowing disorders. A respiratory failure occurred on acute bronchiolitis four months later and he died at the age of 14 months.

#### 3.2.2. Patient Number 5

A 11-month-old girl was hospitalized at the pediatric centre for progressively increasing respiratory distress associated with presumed viral infection. She was the second born child of healthy consanguineous parents. The pregnancy was uneventful. Physical examination during admission revealed a rapid breathing, a perioral cyanosis, and intercostal and subcostal retraction. Chest examination revealed no significant heart murmur but she had coarse breathing sounds. The heart had a normal rhythm. There was a peripheral and axial hypotonia. Deep tendon reflexes were conserved and cranial nerves were uninvolved. A roentgenogram of the chest revealed a diffuse infiltrative process involving the left lung field, but the ECG was normal. CK rate was 3 times the normal value and AST and ALT were 2 times the normal value, and the EMG was compatible with a myopathic disorder. PD was confirmed by deficient activity of acid-glucosidase (GAA) and the genetic studies revealed a homozygous intronic mutation (c.2799 +5 G>A) located outside the consensus splice sites and that has never been described. Her response to antibiotic therapy was temporary and she died of pulmonary failure 15 days after her hospitalization.

#### 3.2.3. Patient Number 6

A 5-month-old boy, the only son of consanguineous parents, was admitted at the pediatric centre because of heart failure due to a hypertrophic cardiomyopathy. After birth, feeding and swallowing difficulties were noticed by his parents. Physical examination upon admission revealed a mild cyanosis of the extremities and intercostal and subcostal retraction. We also noted generalized weakness associated with axial hypotonia and macroglossia. Deep tendon reflexes were abolished. Developmental milestones were delayed; the child could not support his head's weight. Abdominal ultrasound was in favor of moderate hepatomegaly. Chest films showed cardiomegaly with a cardiothoracic ratio of about 0, 78%. CK, ALT, and AST rates were normal. Quantitative blood alpha-glucosidase's level was null. Genetic studies revealed missense mutation L328P that has never been described. He was treated with IV alglucosidase alfa (Myozyme), biweekly but he died at the age of 11 months following respiratory failure that occurred on acute bronchiolitis.

## 4. Discussion

Pompe's disease is a progressive, debilitating, and often fatal neuromuscular disorder resulting from the deficiency of a lysosomal enzyme, acid alpha-glucosidase (GAA) [26]. The disease presents as a continuous clinical spectrum, although it can be broadly classified into IOPD and LOPD forms, according to the age of onset [27]. The IOPD form is the most aggressive and life-threatening form of the disease. Our results confirm and expand on earlier reports documenting the rapid progression and fatal course of this form. All our patients with EOPD presented a typical clinical course and the phenotype was homogenous; they usually present with symptoms within the first months of life and have a rapidly progressive disease course that was usually fatal by one year of age [28]. Generalized hypotonia was a universal feature and our 3 patients exhibited the pathognomonic frog like position. Heart involvement is frequent in IOPD and most infants developed massive and progressive cardiomegaly before age of 6 months [29–31]. Marked cardiomegaly and cardiomyopathy with progression to cardiac failure were observed in 2 of our 3 patients with EOPD. All our patients died during follow-up, and the median age at death was 12 months; cause of death was usually ascribed to cardiorespiratory complications, as reported elsewhere [27]. Other common features were observed, including alert look, hepatomegaly, and macroglossia which were seen in most of our infantile cases [32]. In comparison, patients with LOPD have a less rapid and more variable disease course, where symptoms may begin anywhere from infancy to adulthood. The age at the symptom onset, rate of progression, and sequence of respiratory and skeletal muscle involvement vary by each patient [33]. Our data correlates with the similar age of onset reported by the other studies [9, 34], but the age at diagnosis was considerably older than that in the previous studies. Lack of awareness and qualified diagnostic tests might be the reason for the late diagnosis [27]. The clinical presentation of our patients with LOPD was classical, characterized by progressive limb-girdle muscle weakness in all cases and diaphragm involvement in 2 patients, leading to respiratory insufficiency and early death in the patient number 2 in agreement with previous studies [19, 35]. Progressive dysfunction of the diaphragm and ancillary muscles is the major cause of respiratory insufficiency in children and adults, leading to low lung capacity, blood gas abnormalities, and sleep-disordered breathing. In addition, children and adults may have recurrent respiratory infections due to impaired cough and ineffective airway clearance [34]. In some rare cases, respiratory muscle involvement with respiratory failure as the first and presenting symptom occurs [36]. Death in that case results from cardiorespiratory complications [19]. Progressive weakness of the proximal lower limb and paraspinal muscles contributes to difficulties when walking, running, climbing stairs, and doing other functional activities. Increasing weakness eventually leads to wheelchair dependency [34]. In our series two patients (patient numbers 1 and 2) lost ambulation at the age of 27 and 25 years, respectively. Patient number 3 has experienced mild muscle symptoms since childhood and he is still ambulatory. Many patients eventually require the use of a wheelchair [37]. The clinical symptoms of PD show a significant variability depending on the age at onset, extent of organ involvement, and rate of progression [1, 10, 32]. PD should be considered as part of the differential diagnosis for all children and adults presenting with limb-girdle muscle weakness and respiratory insufficiency without significant cardiomyopathy [8, 21, 38, 39], but cardiomyopathies has also been reported in some cases; it includes rhythm disturbances, such as Wolff–Parkinson–White syndrome, electrocardiogram (ECG) abnormalities, and ascending aorta dilation [37, 40]. In contrast to patients with other neuromuscular disorders, LOPD patients who are still ambulatory may have respiratory insufficiency, which can go undiagnosed [41]. Patients should be specifically asked about nocturnal restlessness, frequent arousals, snoring, excessive daytime sleepiness, headache, and morning lethargy [42]; all these symptoms can orient to respiratory disorders as reported in patient number 1 who presented headaches when waking up. The diagnosis of PD is simple if several manifestations are present but it may be tricky in the presence of isolated symptoms. This variability in disease severity is more probably explained by a different genetic background, because the median age at the time of data collection is approximately the same in different populations [19]. Additional level of residual GAA activity is considered to be associated with severity and rate of disease progression [11]. Typically, infants with little to no detectable enzyme activity (<1%) have a rapid and uniformly fatal disease course. Children and adults with low to moderate GAA activity (1–30%) generally have a less rapid and more variable disease course. Diagnosis of PD must be confirmed by biochemical testing. Diagnosis of PD is confirmed through quantitative enzyme GAA activity assay in tissue samples, including blood, skin fibroblasts, and muscle [20]. New methods have been developed that assay GAA activity in DBS extracts [43]. These novel DBS methods not only are suitable for newborn screening of PD, but also offer a rapid, noninvasive diagnostic test. Muscle biopsy in PD shows the presence of vacuoles that stain positively for glycogen. Given the high anesthesia risk in these patients, muscle biopsies to make a diagnosis of infantile PD should be avoided [30]. In our study, CK levels were increased in 5/6 of our patients, according to the literary data [19], but their elevation is a nonspecific marker for PD [26]. AST and ALT rates were elevated in 4 patients; they reflect enzymes released from muscle [44]. Multiple types of mutations in the GAA gene (locus on 17q23) have been reported worldwide [45]. PD is caused by mutations in the human gene GAA which is located on chromosome 17 and is transcribed into three RNA isoforms encoding the same protein. The gene contains 20 exons (transcript variant 1, NM_000152), and the first exon is noncoded [46, 47]. The cDNA for GAA is greater than 3.6 kb in length width 2856 nucleotides of coding sequence and the resulting product is a protein of 952 amino acids [48]. More than 393 variations have been described in the GAA gene, 257 of which have been confirmed to be pathogenic (www.pompecenter.nl) [27]. Most mutations are extremely rare and limited to individual patients, but common mutations in some populations have been reported. In our study, we identified 3 novel allelic variants predicted to encode truncated forms of GAA: the homozygous intronic mutation (c.2799 +5 G>A), P. Tyr543Asp or p. y543D (c. 1627T>G) and the missense L328P mutation. The effect of the 3 novel mutations should be assessed by functional studies. In 2006, the Food and Drug Administration approved an enzyme replacement therapy (ERT), alglucosidase alfa (Myozyme), for the treatment of PD [22, 42]. The recommended dosage regime of Myozyme ranges from 10 mg/kg/week to 20–40 mg/kg every two weeks, administered as an intravenous infusion. Initial reports of its usage suggest that the ERT has been well tolerated and an improvement in cardiac size and function, as measured by left ventricular mass index (LVMI) and fractional shortening, with a continued improvement in motor function has been recorded [49]. This product was commercialized in Algeria since 2007 and 5 out of 6 of our 6 patients received Myozyme. The treatment was well tolerated by all patients. In clinical studies, the majority of patients developed IgG antibodies to Myozyme, typically within 3 months of treatment. Thus, seroconversion is expected to occur in most patients treated with Myozyme. A tendency was observed for patients treated with a higher dose of Myozyme (40 mg/kg) to develop higher titers of antibody [50]. Our patients should be tested for IgE antibodies and antibody titers ought to be regularly monitored.

## 5. Conclusion

Pompe's disease is now being increasingly diagnosed, due to definitive enzyme estimation facilities. The disease presents as a broad spectrum of clinical phenotypes, with varying rates of progression, symptom onset, degree of organ involvement, and severity. Diagnosis of PD can be accomplished by a simple blood test. With the recent availability of enzyme replacement therapy with Myozyme, the prognosis is likely to change for the better. Gene replacement therapy will eventually be the cure for Pompe's disease and other rare metabolic disorders.

## Figures and Tables

**Table 1:** Demographic characteristics of the study population.

Demographic characteristics of the study population	Case 1	Case 2	Case 3	Case 4	Case 5	Case 6
Age at presentation	31 years	27 years	35 years	5 months	11 months	5 months
Symptomatic since	12 years	21 years	5 years	4 months	11 months	Birth
Sex	Male	Female	Male	Male	Female	Male
Consanguinity	Present	Present	Present	Present	Absent	Present
Motor delay	Absent	Absent	Absent	Absent	Absent	Present
Generalised hypotonia	Absent	Absent	Absent	Present	Present	Present
Hepatomegaly	Absent	Absent	Absent	Present	Absent	Present
Cardiomegaly	Absent	Absent	Absent	Present	Absent	Present
